# Scramblases as Regulators of Proteolytic *ADAM* Function

**DOI:** 10.3390/membranes12020185

**Published:** 2022-02-04

**Authors:** Karina Reiss, Sinje Leitzke, Jana Seidel, Maria Sperrhacke, Sucharit Bhakdi

**Affiliations:** 1 Department of Dermatology, University of Kiel, 24105 Kiel, Germany; sleitzke@dermatology.uni-kiel.de (S.L.); jseidel@dermatology.uni-kiel.de (J.S.); msperrhacke@dermatology.uni-kiel.de (M.S.); 2 Independent Researcher, 24105 Kiel, Germany; Bhakdi_Sucharit@protonmail.com

**Keywords:** *ADAM17*, *ADAM10*, activation, cell membrane asymmetry, phosphatidylserine, scramblases

## Abstract

Proteolytic ectodomain release is a key mechanism for regulating the function of many cell surface proteins. The sheddases *ADAM10* and *ADAM17* are the best-characterized members of the family of transmembrane disintegrin-like metalloproteinase. Constitutive proteolytic activities are low but can be abruptly upregulated via inside-out signaling triggered by diverse activating events. Emerging evidence indicates that the plasma membrane itself must be assigned a dominant role in upregulation of sheddase function. Data are discussed that tentatively identify phospholipid scramblases as central players during these events. We propose that scramblase-dependent externalization of the negatively charged phospholipid phosphatidylserine (PS) plays an important role in the final activation step of *ADAM10* and *ADAM17*. In this manuscript, we summarize the current knowledge on the interplay of cell membrane changes, PS exposure, and proteolytic activity of transmembrane proteases as well as the potential consequences in the context of immune response, infection, and cancer. The novel concept that scramblases regulate the action of *ADAM*-proteases may be extendable to other functional proteins that act at the cell surface.

## 1. Introduction

Membrane anchored metalloproteases of the *ADAM* family assume central functions in the living cell by the controlled cleavage and release of biologically active proteins and peptides from the membrane surface. Two predominant members, *ADAM10* and *ADAM17*, are indispensable for embryonic development in mice [1,2]. Loss of ADAM17 is associated with severe multiorgan dysfunction in humans. Patients with a homozygous mutation in *ADAM17* presented with severe diarrhea, skin rash, and recurrent sepsis [3,4,5].

*ADAM17* was originally identified as the TNF-alpha releasing enzyme [6,7]. Today, *ADAM17* is known to be involved in the shedding of an increasing number of cell surface proteins including the EGFR ligands TGF-α and amphiregulin (AREG), TNF receptor 1, and L-selectin. Very diverse biological processes are thus regulated by a single protease. 

*ADAM10* is the major sheddase of cell adhesion molecules including neuronal (N)-cadherin [8], epithelial (E)-cadherin [9], and vascular-endothelial (VE)-cadherin [10], but also releases the EGFR ligands betacellulin (BTC) and EGF [11] and the low affinity IgE receptor CD23 [12]. Moreover, the protease mediates the non-amyloidogenic α-secretase cleavage of the Alzheimer’s precursor protein. Dysregulated *ADAM10* activity is assumed to play a central role in diverse pathologies including Alzheimer’s disease, allergic responses, and cancer development [13,14].

The bewilderingly wide spectrum of potential substrates on the one hand is matched by the complexity of cellular processes that fine-tune the individual shedding events on the other. The post-translational regulation of *ADAM10* and *ADAM17* sheddase activity is multifaceted. For *ADAM17*, inactive rhomboid proteins, iRhom1 and iRhom2, are assumed to be key regulators of maturation, protease function and substrate selectivity [15,16,17]. Tetraspanins play an important role for *ADAM10* subcellular localization and substrate interaction [18,19]. Moreover, sheddase activity is modulated by changing interaction partners as well as subcellular compartmentalization [20]. 

A remarkably broad and heterogeneous spectrum of stimuli has been found to activate the enzymes [21,22,23,24], whereupon substrate cleavage occurs at sites located very close to the cell membrane surface.

Functional upregulation of *ADAM10* is generally observed in association with cytosolic Ca^2+^ elevation—as elicitable by treatment of cells with Ca^2+^ ionophores, purinergic receptor agonists, or membrane-perturbating agents [23,25,26]. *ADAM17* sheddase activity is amplified by more diverse signaling pathways including activation of protein kinase C (PKC) and tyrosine kinases such as VEGFR2 or EGFR [27]. 

The main thrust of research into the control of sheddase activation has been conducted on these two proteases. They have targeted dissection of events underlying the trafficking of the proteases to the cell surface, and of regulatory roles assignable to the extracellular domains of the proteases [28,29]. The present review introduces a novel aspect into the discussion. We summarize current knowledge regarding the significance of PS externalization on proteolytic activity of *ADAM10* and *ADAM17*. Arguments are presented to support the concept that scramblase-mediated shuffling of phospholipids is a key step leading to *ADAM10* and *ADAM17* activation [30,31,32,33,34,35]. The potential functional consequences of these interactions are discussed and future challenges to be met in field are outlined.

## 2. *ADAM*s and the Cell Membrane

The multifaceted role of the cell membrane in the regulation of shedding has been an emerging theme in recent years [26,36]. Cell membrane fluidity appears to directly promote substrate–protease interaction. Fluidity is affected by content of cholesterol and unsaturated free fatty acids (FFA). Membrane cholesterol depletion led to increased sheddase activity, as did the enhancement of lateral protein mobility evoked through incorporation of unsaturated FFA [37]. 

Organization of membrane nanostructure is a second major issue. Nanodomains rich in cholesterol and sphingolipids are thought to form platforms for substrate–protease interaction in the plasma membrane. Depletion of cholesterol or sphingomyelin enhances shedding of several *ADAM* substrates. Application of sphingomyelinase leads to formation of ceramide-enriched nanodomains. This resulted in increased *ADAM17*-mediated release of substrates in different cells [24]. 

Increasing evidence indicates that assembly in multiprotein complexes modulates *ADAM* locations and thus discriminates substrate specificity as well as timing of sheddase activation. Vesicular compartments and intracellular organelles work as structural scaffolds to coordinate specificity and temporal activity of functional hubs in cell signaling and enzymatic function [20]. 

## 3. PS Exposure and Scramblases

### 3.1. Cell Membrane Asymmetry

A germane property of common *ADAM* stimuli is the breakdown of phospholipid asymmetry. The non-random distribution of different lipid species in the lipid bilayer is a common feature of all eukaryotic membranes. Phosphatidylserine (PS) is exclusively located in the inner leaflet while phosphatidylcholine (PC) and glycolipids are mainly located in the outer leaflet of the membrane. This asymmetry is maintained by P4-type ATPases (flippases), which transfer the amino phospholipids PS and phosphatidylethanolamine (PE) to the cytoplasmic membrane leaflet [38,39].

Irreversible PS externalization occurring upon ATP depletion is a key signal for apoptotic cell clearance. Massive PS externalization in activated platelets triggers blood and platelet coagulation [40]. 

Less well known is the fact that breakdown of membrane asymmetry also occurs as a transient event in many physiological situations. Among others, surface PS exposure is involved in myoblast and osteoclast fusion and is critical for neuronal regeneration [40].

PS is externalized through the activation of scramblases, proteins that non-specifically and bidirectionally translocate phospholipids between the outer and inner leaflets of the plasma membrane. The existence of scramblases was postulated decades ago, but their molecular identity eluded definition until recently. Diverse transmembrane proteins have been implicated in lipid scrambling. Members of the TMEM16 family are by far the best characterized [41,42].

### 3.2. The TMEM16/Anoctamin Family

There are 10 human TMEM16/Anoctamin (ANO) proteins. Although structurally related, TMEM16A/ANO1 and TMEM16B/ANO2 function as Ca^2+^-activated chloride channels and lack scramblase activity. Mammalian TMEM16C/ANO3, D/ANO4, E/ANO5, G/ANO7, and K/ANO10 are primarily assigned scramblase activity, while TMEM16F/ANO6 and TMEM16J/ANO9 apparently fulfil dual functions as cationic channels and scramblases [41,43,44]. The function of TMEM16H/ANO8 has not yet been elucidated. ANO-provoked breakdown of cell membrane phospholipid asymmetry can trigger a plethora of cellular responses such as blood coagulation [45,46], microparticle release [47], membrane repair [48], cell–cell fusion [49,50,51,52], and viral infection [40,53,54]. Malfunctions in TMEM16/ANO proteins have been implicated in human diseases, including asthma, cancer, bleeding disorders, muscular dystrophy, arthritis, epilepsy, dystonia, and ataxia [55,56,57,58].

### 3.3. Xkr Scramblases

Scramblase activity at the plasma membrane was also attributed to members of the Xkr family which comprises nine family members in humans. Xkr8 was shown to facilitate PS exposure in apoptotic cells by a mechanism that involves cleavage by caspases or activation via phosphorylation near the caspase recognition site [59,60]. Apoptotic Xkr8-deficient cells do not expose PS. After transfection with Xkr4, Xkr8, or Xkr9, they responded to apoptotic stimuli with PS exposure at the cell surface [59]. However, the question whether these proteins act as bona fide lipid scramblases is still a matter of debate since studies with purified proteins reconstituted into synthetic vesicles have yielded contradicting results with XKR9 [61] and are not available for XKR4 and XKR8.

### 3.4. Additional Phosphlipid Scramblases

There is a third family of phospholipid-translocating proteins, designated phospholipid scramblases (PLSCR). Four human members of this family have been identified hPLSCR1-4 [62]. hPLSCR1 and hPLSCR3, the most extensively studied proteins in this family, are reported to have crucial roles in apoptosis. Recombinant purified hPLSCR1, hPLSCR3 and hPLSCR4 showed scrambling activity in vitro when reconstituted in proteoliposomes [63], but the true in vivo role of hPLSCRs in PS exposure still remains a matter of debate [40,64,65].

In addition, some other transmembrane proteins have recently been implicated in lipid scrambling such as few G protein-coupled receptors, the autophagy protein Atg9, and the ER protein complex TMEM41B/VMP1 [66,67,68].

## 4. The Link between Sheddase and Scramblase Activity

### 4.1. PS Exposure and ADAM17 Activity 

Scott syndrome is a rare bleeding disorder caused by the incapacity of blood cells to expose PS in response to intracellular Ca^2+^ elevation. The defect is due to a missense mutation in the calcium-dependent PS scramblase ANO6 [45,69]. The link between the function of sheddases and scramblases was initially uncovered through experiments with lymphocytes from Scott syndrome patients. Calcium influx provoked rapid PS exposure and loss of the *ADAM17* substrate L-selectin in normal B-cells, but Scott lymphocytes responded neither with PS exposure nor with substrate shedding. Expression of caspase-dependent scramblases is unaltered in Scott lymphocytes, so the decisive experiment was performed to examine whether apoptosis induction would provoke normal PS-externalization and shedding of the *ADAM17* substrate. This turned out to be the case, and the possible mechanism underlying *ADAM17* activation by PS was investigated [35]. If PS directly interacted with the protease, its soluble head group *ortho*-phosphorylserine (OPS) would possibly act as a competitive inhibitor. Indeed, *ADAM17*-dependent substrate shedding was found to be reduced in the presence of OPS in several cell models.

**Figure 1:**
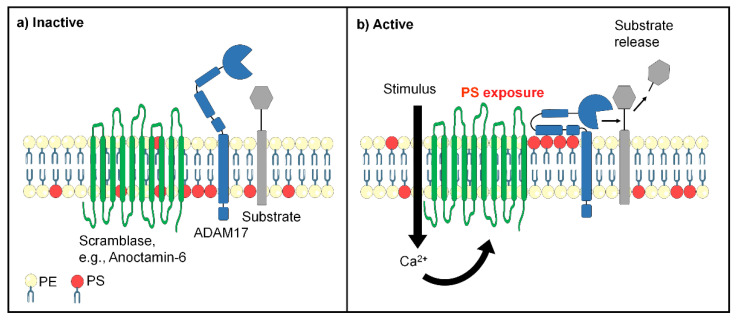
Proposed links between scramblase and *ADAM17* function. (**a**) In non-activated cells, negatively charged phosphatidylserine (PS, red) is mainly sequestered in the inner cell membrane leaflet, while phosphatidylcholine (yellow) is mainly localized in the exoplasmic leaflet. *ADAM17* (blue) has limited access to substrates. The ectodomain consists of a metalloprotease domain, a disintegrin domain, and a membrane-proximal domain (MPD) followed by a stalk region. (**b**) Cell stimulation can lead to scramblase activation and rapid loss of cell membrane asymmetry. Externalized PS electrostatically interacts with positively charged amino acids of *ADAM17* and guides the enzyme to its substrate.

This prompted a search for a PS interaction motif in the ectodomain of the protein. Commencing at the membrane surface, the ectodomain comprises a stalk region, a membrane-proximal domain (MPD), a disintegrin-like domain, and the catalytic domain with resolved crystal structure [70,71] (Figure 1). The stalk region of *ADAM17* contains a unique evolutionally conserved sequence called CANDIS (Conserved Adam seventeeN Dynamic Interaction Sequence), which forms an amphipathic helix that can interact with the cell membrane [72].

The MPD represented a likely candidate for interaction with PS because of its proximity to the membrane surface. To test for this possibility, recombinant MPD was produced and found to bind to PS but not to PC liposomes. NMR-spectroscopy localized the PS interaction site to a cluster of basic amino acids, R625/K626/K628. Mutation of these amino acids to glycines abolished PS binding capacity. When the corresponding *ADAM17* mutant was transfected into *ADAM10*/*ADAM17*-double deficient cells, it was no longer able to cleave its physiological substrate TGF-α. However, the cells did express *ADAM17* on their surface and the mutated protease was still capable of cleaving a soluble peptide substrate in the culture medium. A key finding was thus made that abrogation of PS binding selectively affected the release of cell membrane-bound substrates but not the bona fide enzymatic activity of the protease (Figure 1). 

The relevance of these findings was confirmed in vivo. Mutagenesis of the three amino acids constituting the PS-binding motif led to embryonic lethality in mice [32]. Primary hepatocytes and fibroblasts were found to express the mutant protease on the cell surface. However, release of *ADAM17* substrates was completely abolished. The results directly supported the concept of transiently externalized PS as the essential trigger of *ADAM17* sheddase activity in vivo.

Further studies bore out the contention that ANO6 is a key regulator of *ADAM17* function [30]. Overexpression of ANO6 in HEK293T cells led to increased Ca^2+^-mediated PS exposure that was accompanied by enhanced release of *ADAM17* substrates. Transfection of cells with a constitutively active ANO6 mutant led to spontaneous PS exposure and to enhanced release of *ADAM17* substrates in the entire absence of any stimuli. Inhibitor experiments indicated that ANO6-mediated enhancement of substrate cleavage simultaneously broadened the spectrum of participating metalloproteinases. Complementary experiments showed that siRNA-mediated downregulation of ANO6 in human umbilical vein endothelial cells decreased ionophore-mediated release of TNFR1. 

### 4.2. ADAM10 Sheddase Function and PS Externalization

The question arose whether the homologous protease *ADAM10* would similarly be subject to regulation by ANO6 and PS exposure.

Our results pointed to such a scenario [31]. Overexpression of ANO6 led to increased PS externalization and substrate release. Transfection with a constitutively active form of ANO6 resulted in maximum sheddase activity in the absence of any stimulus. Calcium-dependent *ADAM10* activation could not be induced in lymphocytes of patients with Scott syndrome harboring a missense mutation in ANO6. In principle analogy with *ADAM17*, inhibition experiments with soluble OPS indicated that triggering of proteolytic activity involved a direct interaction of surface-exposed PS with the protein. *ADAM10* has basically a similar modular structure as *ADAM17*. The X-ray crystal structure of the *ADAM10* ectodomain has been elucidated by Seegar et al [73]. It was found that the enzyme active site is occluded by a short peptide loop located at the commencement of the stalk region (residues 647–655). We became aware that a putative PS binding site similar to the cationic motif identified in *ADAM17* follows immediately after this inhibitory loop (residues 657/659/660) within the *ADAM10* stalk region. Alteration of this motif abrogated sheddase activation by externalized PS [31]. A simple model evolved in which surface-exposed PS attracts and draws this peptide sequence down to the membrane surface. As a result, the enzyme-inhibiting loop will be drawn out of the catalytic site which can then access its intended substrate [31].

## 5. *ADAM*s and Scramblases in Health and Disease

### 5.1. Immune Responses

**Figure 2:**
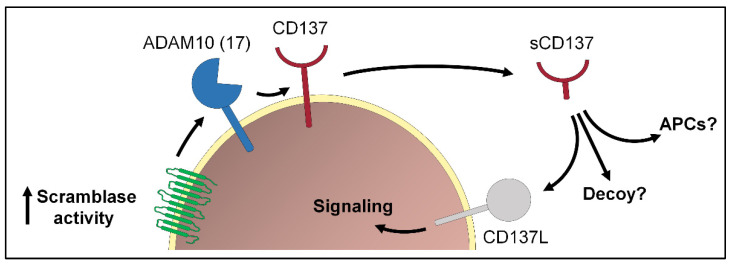
Transient exposure of PS plays an important role in the immune system. One example is the release of the TNFR family member CD137 via *ADAM10* (or *ADAM17*). Soluble CD137 (sCD137) can bind to its ligand CD137L expressed on activated T cells and activate cell signaling. Whether sCD137 could fulfill additional functions, e.g., activation of antigen-presenting cells (APCs) or act as decoy is still not clear.

Transient PS exposure is integral to a multitude of activation events in cells of the immune system, although the relevance thereof remains unclear in many instances. Such is the case with activated neutrophils [74]. In mast cells, transient exposure and cell degranulation are co-induced by IgE receptor stimulation [75]. Transient PS externalization has also been described in T cells. Elliott et al. identified a role for PS distribution changes in signal transduction, rapidly modulating the activities of several membrane proteins including the P2X_7_ cation channel [76]. P2X_7_-stimulated transient PS externalization induced shedding of the homing receptor L-selectin in T cells. In macrophages, ANO6 could be identified as a responsible scramblase and essential component of innate immunity downstream of P2X_7_ [77]. A new link was recently uncovered between an immunological axis and the function of sheddases. CD137 is a member of the TNFR family that functions as costimulatory molecule, promoting proliferation and survival of activated T cells. A soluble form of CD137 (sCD137), hitherto considered to represent a splice variant of the membrane-anchored molecule, circulates and is elevated in plasma of patients with rheumatoid arthritis and diverse malignancies [78,79,80]. A directed search led to the finding that *ADAM10* is centrally involved in the generation of sCD137 [33]. Release of sCD137 was markedly suppressed when *ADAM10* sheddase function was inhibited by either conventional inhibitors or through the presence of soluble phosphorylserine. Overexpression of ANO6 increased stimulated shedding, and hyperactive ANO6 led to maximal constitutive shedding of CD137. sCD137 was functionally active and augmented T cell proliferation (Figure 2). The collective findings potentially impact current immunotherapeutic approaches that are targeting CD137 in a variety of diseases.

### 5.2. Cancer

TMEM16 proteins are associated with diverse malignancies. Overexpression correlates with poor prognosis in breast, head and neck, and pancreas cancer [39,81]. Inhibition of TMEM16A/ANO1 function reportedly suppresses cancer cell proliferation and migration [82,83]. TMEM16D/ANO4 has been associated with breast cancer [84,85]. TMEM16G/ANO7 is upregulated in prostate cancer [86]. TMEM16J/ANO9 was linked to pancreatic and colorectal cancer [87,88]. In pancreatic cancer it supposedly promotes tumorigenesis via modulation of EGFR signaling. This equates with a direct link to *ADAM10* and *ADAM17*, which are the major sheddases of EGFR ligands. In an immediate context, both *ADAM*s play a profound role in many types of cancers [89,90]. Recent results from our working group indicate a direct link between ANO4 and ANO9 scramblase activity and *ADAM* function [34]. Overexpression of ANO4 and ANO9 led to increased release of *ADAM10* and *ADAM17* substrates, such as betacellulin, TGF-α, and AREG, upon ionophore stimulation in HEK cells. Increased PS exposure was observed under constitutive as well as under stimulated conditions. The direct link between scramblase activity and *ADAM* activity emerged in competition experiments with the soluble PS headgroup phosphorylserine. Overexpression of ANO4 or ANO9 in human cervical cancer cells (HeLa) enhanced constitutive shedding of the growth factor AREG and increased cell proliferation. These data indicate that ANO4 and ANO9, by virtue of their scramblase activity, may play a role as important regulators of *ADAM*-dependent tumor cell functions. Uncovering the detailed connections between TMEM proteins and *ADAM*s in cancer will become a rewarding field of cancer research in the foreseeable future. 

Another interesting aspect of the role of *ADAM*s and scramblases in cancer concerns the role of extracellular vesicles (EVs). Released exosomes are present in body fluids including blood or bronchoalveolar fluid, and this release is increased in many pathologies ranging from oncogenesis to inflammation [91,92]. Cancer cell released exosomes play an important role in promoting progression of cancers by increasing their invasive potential [93]. They are carried through the blood and lymph circulation and affect the development of the primary tumor as well as distant metastasis through the transfer of RNA and proteins [94]. Externalization of PS and PE alters lipid packing in the membrane and influences the membrane curvature [95]. An important consequence is the release of extracellular vesicles as intercellular messengers. A direct connection of Anoctamins with vesicle and exosome release has been described [81,95]. In particular, a central role for the release of vesicles has been reported for ANO6 [47,96,97] and ANO1 [98]. A similar function has been suggested for ANO7 in the context of prostate cancer [99] that might also apply for other Anoctamin family members. In this context, it is of distinct interest that both *ADAM10* and *ADAM17* are reportedly present in exosomes [20]. It has been shown that exosomal *ADAM10* and *ADAM17* retain their biological activity and enhance substrate release in target cells. Addition of exosomes to cells expressing the *ADAM17* substrates TGF-α and amphiregulin led to enhanced shedding [100]. Notably, contribution of exosomal *ADAM10* activity could also be shown for shedding of the *ADAM10* substrates CD44 and L1 [101,102,103]. Increased ANO scramblase activity could thus enhance *ADAM* activity and the release of, e.g., tumor growth factors on the same cell. In addition, ANO scramblase activity could increase the release of *ADAM*-containing vesicles that could further promote tumor growth in distant target cells. 

### 5.3. Virus and Bacterial Infection

The presence of PS in the target membrane promotes fusion of many enveloped viruses with host cells [39,104,105]. HIV-1 entry into host cells starts with interactions between the viral envelope glycoprotein (Env) and cellular CD4 receptors and co-receptors. Formation of the pre-fusion receptor/co-receptor complexes triggers non-apoptotic cell surface exposure of PS. This event involves activation of the lipid scramblase TMEM16F/ANO6 and depends on Ca^2+^ signaling. Externalized PS promotes Env-mediated membrane fusion and HIV-1 infection. Blockade of externalized PS or suppression of TMEM16F resulted in the inhibition of Env-mediated fusion and infection [53]. Promotion of membrane fusion by surface-exposed PS seems to be relevant for the entry of many other viruses including vesicular stomatitis virus (VSV) or alpha-herpesvirus into the cell [106,107].

Further to allowing viral entry, PS externalization may play a general downstream role because viral replication necessarily involves cell activation events that will involve Ca^2+^ influx. Then, activation of scramblases and sheddases cannot but follow. Perhaps these recent recognitions will provide the speculation with further impetus that targeting exposed anionic phospholipids might protect against lethal virus infections in vivo [105].

The spike protein of SARS-CoV-2 can also activate TMEM16F/ANO6 and thus induce syncytia formation [50], a finding consistent with the previously proposed role of PS exposure in physiologic cell fusion events [40]. Moreover, it has been speculated that PS exposure may be an important mechanism related to *ADAM17*-mediated ACE2, TNF-alpha, EGFR and IL-6R shedding that might contribute to the pathophysiology of COVID-19 inflammation and coagulation abnormalities [108]. However, the possible relevance for the disease is not clear. 

**Figure 3:**
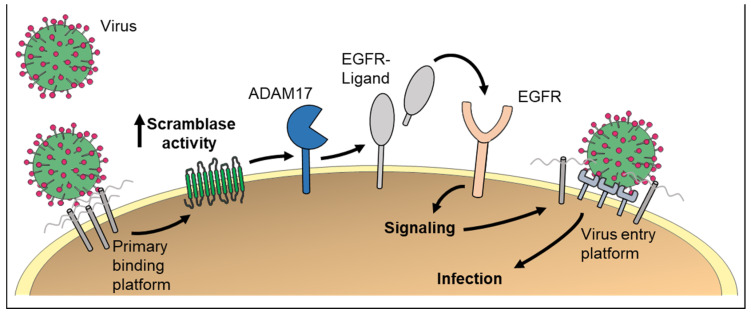
PS externalization could be of high relevance for virus entry into host cells. First, formation of the primary virus–receptor complex triggers non-apoptotic cell surface exposure of PS via scramblases. Externalized PS promotes membrane fusion and virus infection. Second, scramblase activation would lead to *ADAM* activation. Subsequent EGFR signaling has been identified as important step for infection with the human papillomavirus.

PS externalization could promote virus infection in an additional way, namely by activating *ADAM* sheddase function. The importance of *ADAM* activity for viral infections was recently demonstrated for human papillomavirus (HPV) [109]. HPVs are small DNA viruses that infect epithelial cells. After HPV binding to cell surface receptors, a cascade of molecular interactions mediates viral internalization. Metalloproteases of unknown identity appeared to be involved in these processes [110], and we recently identified *ADAM17* as the prime candidate [109]. It was found that shedding of growth factors by *ADAM17* triggered the extracellular signal-regulated kinases (ERK1/2) pathway, which then led to formation of the endocytic entry platform for the virus (Figure 3). In subsequent studies, the tetraspanin CD9 was identified as another regulator of *ADAM17* activity and HPV infection [111]. 

An interesting link to *ADAM10* comes from the field of bacterial defense. *ADAM10* is a high-affinity receptor for cytotoxic *Staphylococcus aureus* alpha-toxin [112]. The protease itself is subject to cleavage and removal from the membrane surface by other sheddases [113]. This must be expected to render the respective cells less susceptible to the action of alpha-toxin, one of the most important pathogenicity factors of *Staphylococcus aureus* [114]. Intriguingly, Lizak and Yarovinsky (2012) have reported that IFNα-mediated protection from alpha-toxin is dependent on induction of PLSCR1 [115]. If increased expression of PLSCR1 would lead to enhanced PS exposure, the activation of transmembrane metalloproteases might reduce the surface amounts of *ADAM10* and limit the cytotoxic effects of alpha-toxin. 

It is evident that we are witnessing just the beginning of an exciting field of research into the interwoven roles of scramblases and sheddases in the context of viral and bacterial infections. 

## 6. Conclusions and Perspectives

Externalization of PS effected by scramblases is envisaged to exert a key regulatory function in controlling substrate cleavage by *ADAM*10 and *ADAM17*. Major challenges arise for future research. There is still little reliable data that indicate whether proposed scramblase proteins indeed function as a scramblase or whether other molecules are necessary. This could only be examined by appropriate in vitro reconstitution assays in synthetic proteoliposomes. In addition, knockout and gain of function mouse models could help to further understand the in vivo relevance and the potential compensation mechanisms. It is obvious that several proteins must be involved in such a central element of life as the regulation cell membrane asymmetry. It is also clear that *ADAM10* and *ADAM17* will not be the only proteins whose function is regulated by scramblases. Our data indicate that ANO6-mediated enhancement of substrate cleavage simultaneously broadened the spectrum of participating metalloproteinases far beyond *ADAM10* and *ADAM17* [30]. In accordance with the literature, cleavage of TGF-alpha provoked by ionophore in normal cells is affected predominantly by *ADAM17* and inhibitable with *ADAM17* inhibitors. In cells overexpressing ANO6, however, the substrate release could not be blocked anymore with *ADAM17* inhibitors but only with broad-spectrum metalloprotease inhibitors, indicating that further metalloproteases participated in the cleavage of TGF-alpha [30]. Recently, we obtained similar results upon ANO4 and ANO9 overexpression [34]. Could other membrane-anchored proteases or proteins operating at the cell surface underlie similar regulatory principles? These and many other intriguing questions await resolution. 

The scramblase–*ADAM* connection could also be important under pathologic conditions. The proteases promote several inflammatory as well as tumorigenic pathways [90,116,117,118]. Much less is known about the significance of the scramblases in health and disease, but there are indications that there may be a causal link to protease activity. To target scramblase proteins and treat scramblase-related diseases, it is critical to have a comprehensive understanding of these proteins and their function at the molecular level. Elucidation of the possible links between scramblase activity and protease/protein function represent an exciting future challenge for research in cell membrane biology.
